# Supplementary material for: MafB is a critical regulator of complement component C1q
Source: Nat Commun. 2017 Nov 22;8:1700. doi: 10.1038/s41467-017-01711-0 (PMC5700178; doi:10.1038/s41467-017-01711-0)
Supplement: Supplementary file 1 — Supplementary Information [file 41467_2017_1711_MOESM1_ESM.pdf]

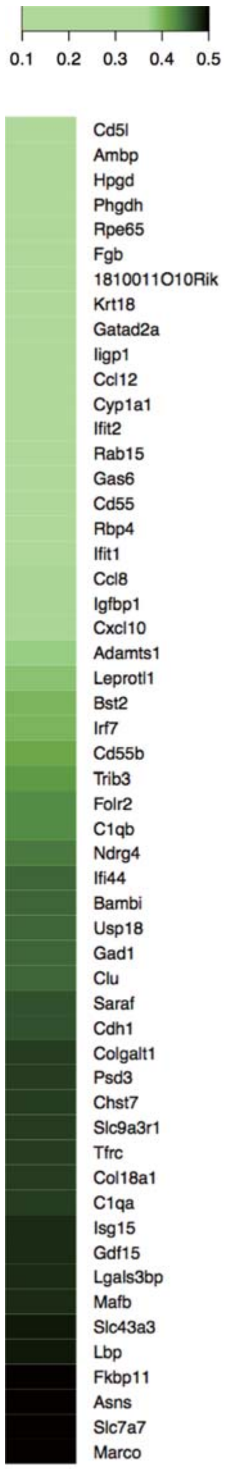

Supplementary Fig. 1. Microarray data analysis of *Mafb*$^{-/-}$ macrophages

Microarray data with a *Mafb*$^{-/-}$/WT signal ratio less than 0.5 from the *Mafb*$^{-/-}$ and WT non-adherent macrophages are shown (data accessible in the NCBI GEO database, accession no. GSE20419).

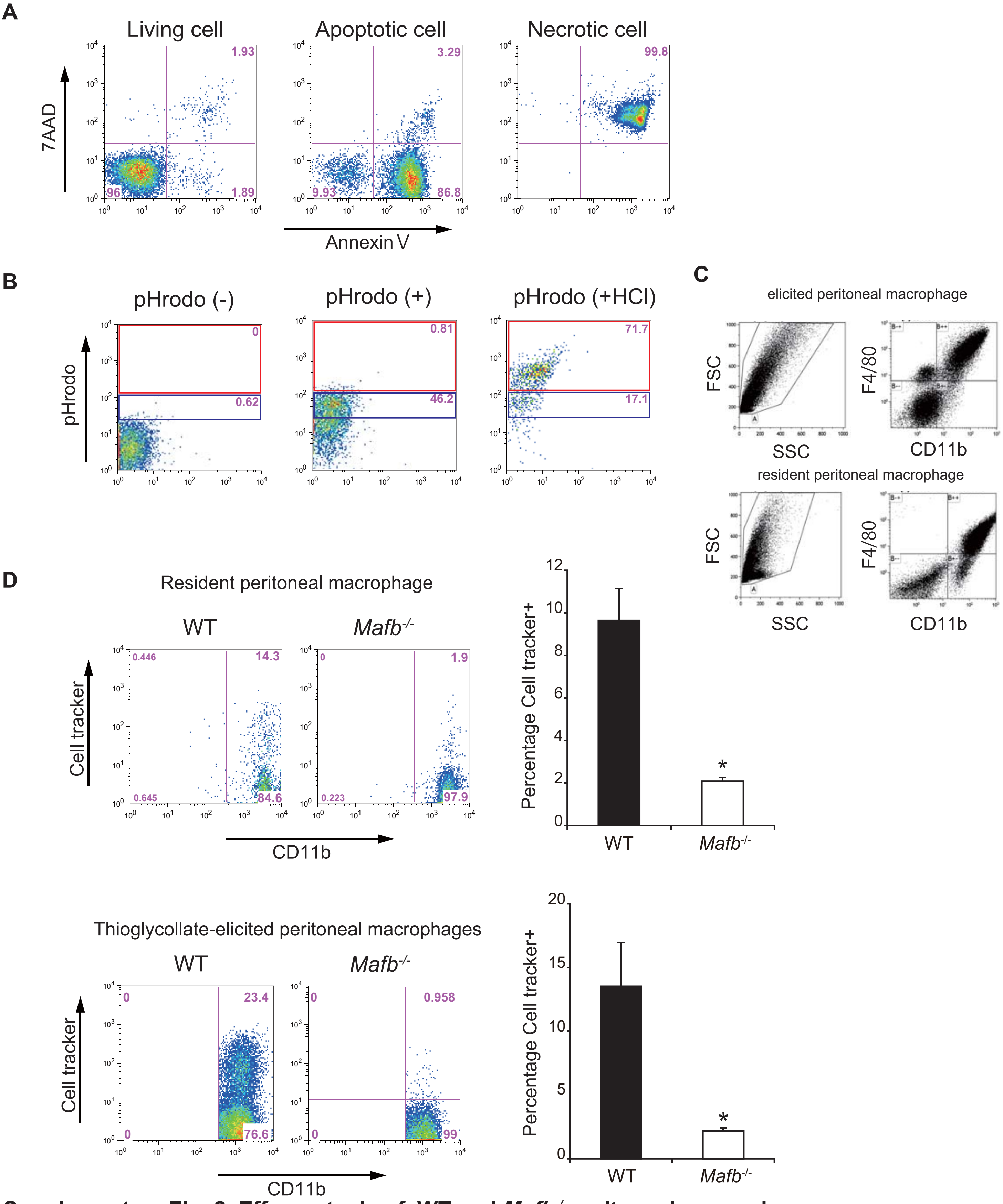

Supplementary Fig. 2. Efferocytosis of WT and *Mafb*$^{-/-}$ peritoneal macrophages
(**A**) Early apoptotic cells were induced with 0.07 μM dexamethasone and cultured for 6 hours. Necrosis was induced by incubating thymocytes at 56 °C for 1 hour. Early apoptotic cells were positive for Annexin V but negative for 7AAD. (**B**) Early apoptotic cells were incubated with pHrodo (20 ng/ml) for 1 hour. The addition of 1 M HCl induced strong fluorescence by pHrodo. (**C**) Peritoneal macrophages (elicited and resident) were stained with F4/80 and CD11b and gated on the left FSC and SSC plots. (**D**) The left panel shows that apoptotic cells treated with CellTracker were injected into the abdominal cavities of WT and *Mafb*$^{-/-}$ fetal liver-transplanted mice. After 30 minutes, cells from the abdominal cavity were analyzed by flow cytometry. The statistical analysis is shown in the right panel. Data are presented as the mean ± s.e.m.; * $p<0.05$ compared with WT (Welch's t-test). The data are from one experiment that is representative of at least two independent experiments.

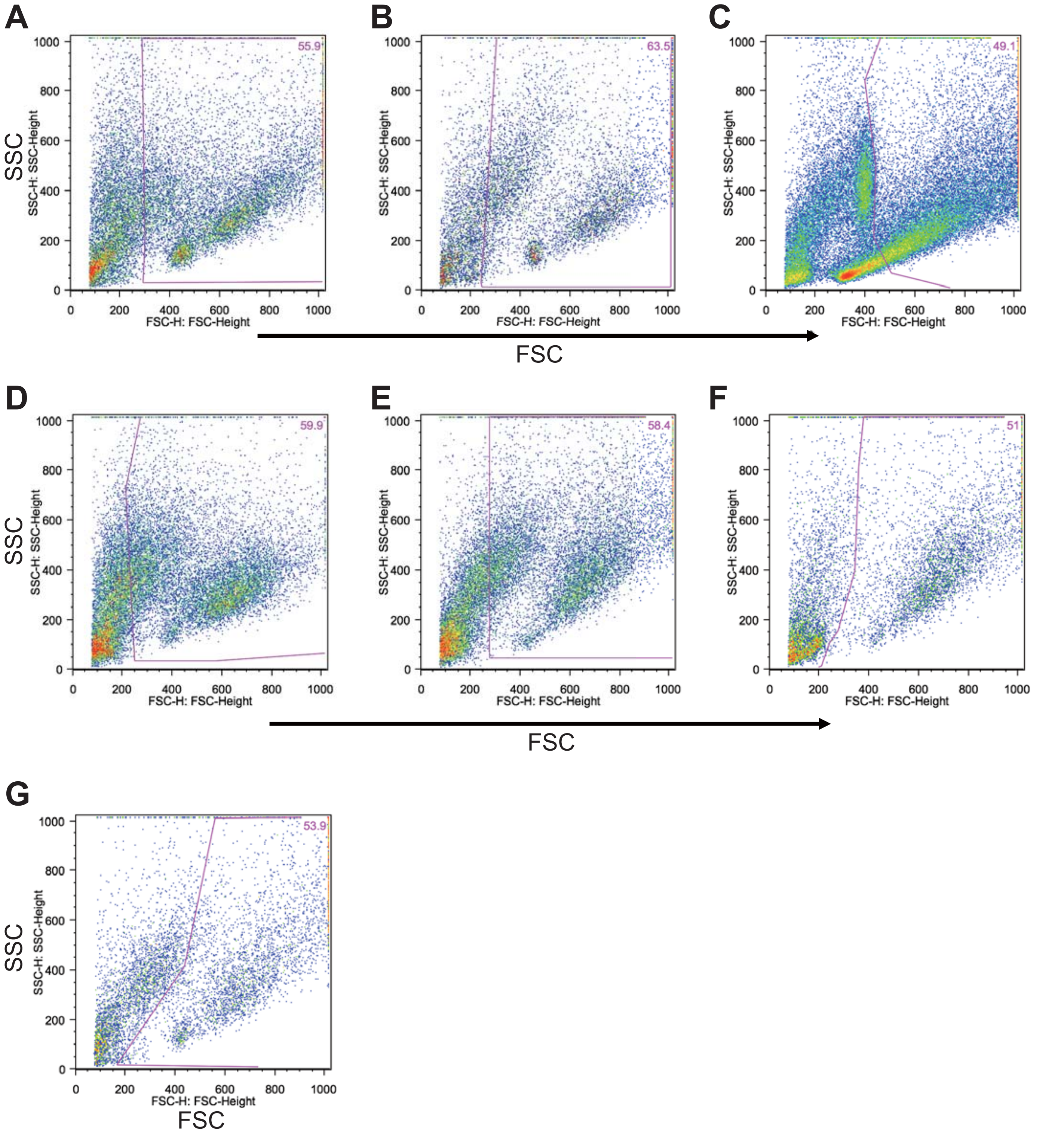

Supplementary Fig. 3. Gating strategy for FACS analysis.
(**A**) Gating strategy for the data in Fig. 1B. (B) Gating strategy for the data in Fig. 1D. (C) Gating strategy for the data in Fig. 1G. (D) Gating strategy for the data in Fig. 4A. (E) Gating strategy for the data in Fig. 4B. (F) Gating strategy for the data in Fig. 4C. (G) Gating strategy for the data in Fig. 4D.

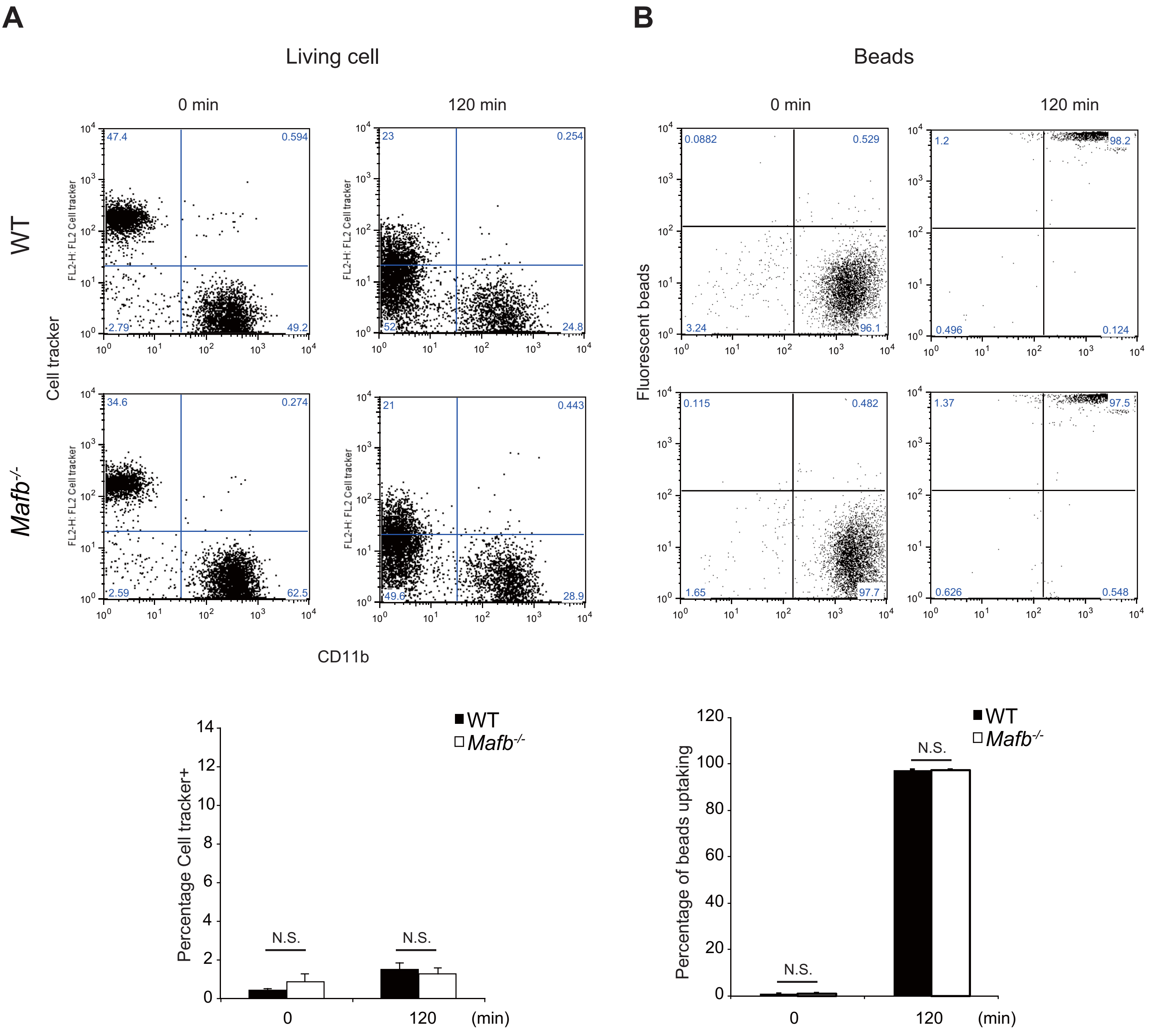

Supplementary Fig. 4. Uptake of living cells and fluorescent beads did not differ between WT and *Mafb*$^{-/-}$ macrophages.

(**A**) Fetal liver-derived macrophages were treated with normal thymocytes. Neither WT nor *Mafb*$^{-/-}$ macrophages could take up thymocytes (WT, n = 4; *Mafb*$^{-/-}$, n = 4). (**B**) Both WT and *Mafb*$^{-/-}$ macrophages could take up fluorescent beads (WT, n = 6; *Mafb*$^{-/-}$, n = 4). (**A**, **B**) Data are presented as the mean ± s.e.m.; N.S., not significant (Welch's t-test).The data are from one experiment that is representative of at least two independent experiments.

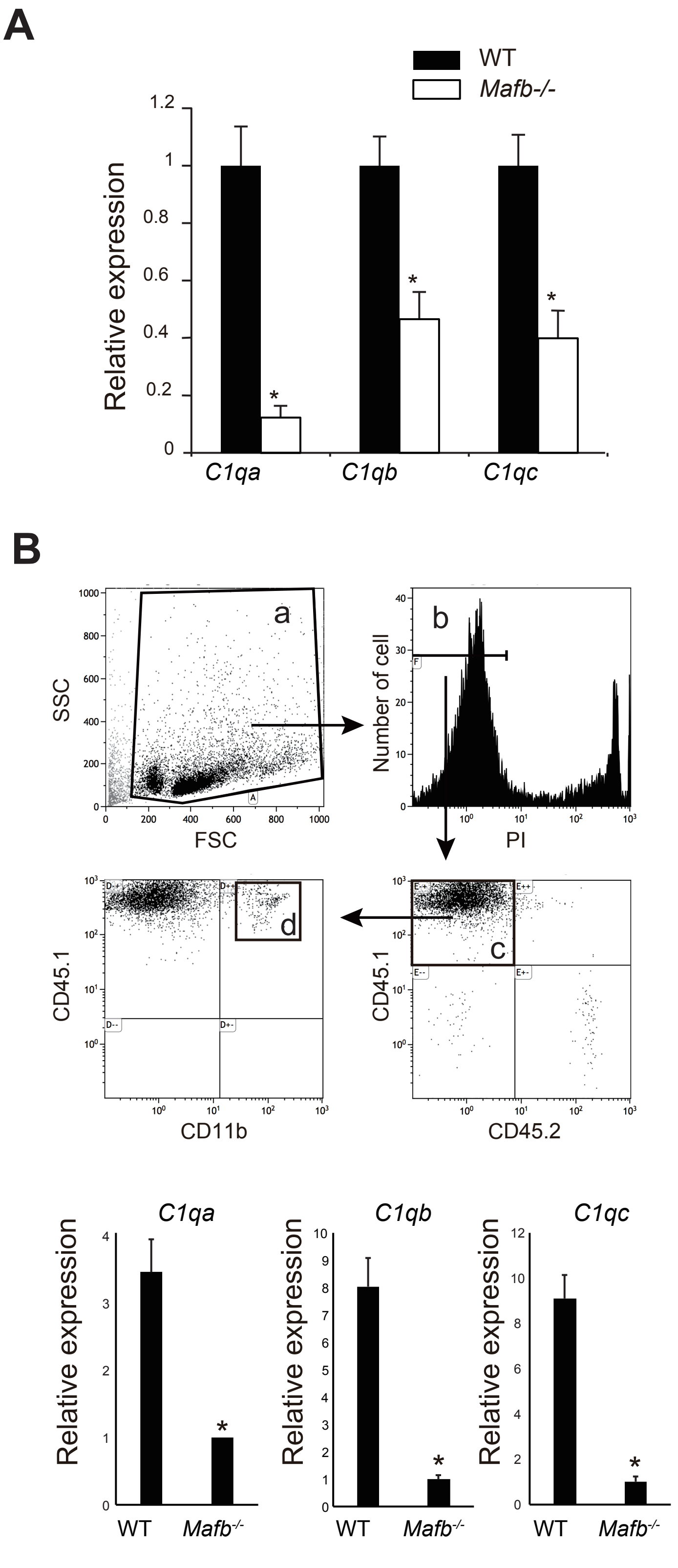

Supplementary Fig. 5. MafB regulated the *C1qa, C1qb, and C1qc* genes.
(**A**) *C1q* genes expression in fetal liver-derived macrophages was analyzed by qRT-PCR (WT, n=15; *Mafb*$^{-/-}$, n=10). * *p*<0.01 compared with WT (Mann-Whitney U-test). The results of 2 independent experiments were pooled. (**B**) CD45.1+CD11b+ macrophages ($2x10^5$) were sorted from the spleens of lethally irradiated mice 6 months after receiving a fetal liver transplants from WT or *Mafb*$^{-/-}$ mice. The upper panel shows the sorting settings using gates a, b, c and d. The lower panel shows the mRNA levels of *C1qa*, *C1qb*, and *C1qc* as measured by qRT-PCR (n = 5 for each sample). * *p*<0.05 compared with WT (Mann-Whitney U-test). The data are from one experiment that is representative of two independent experiments.

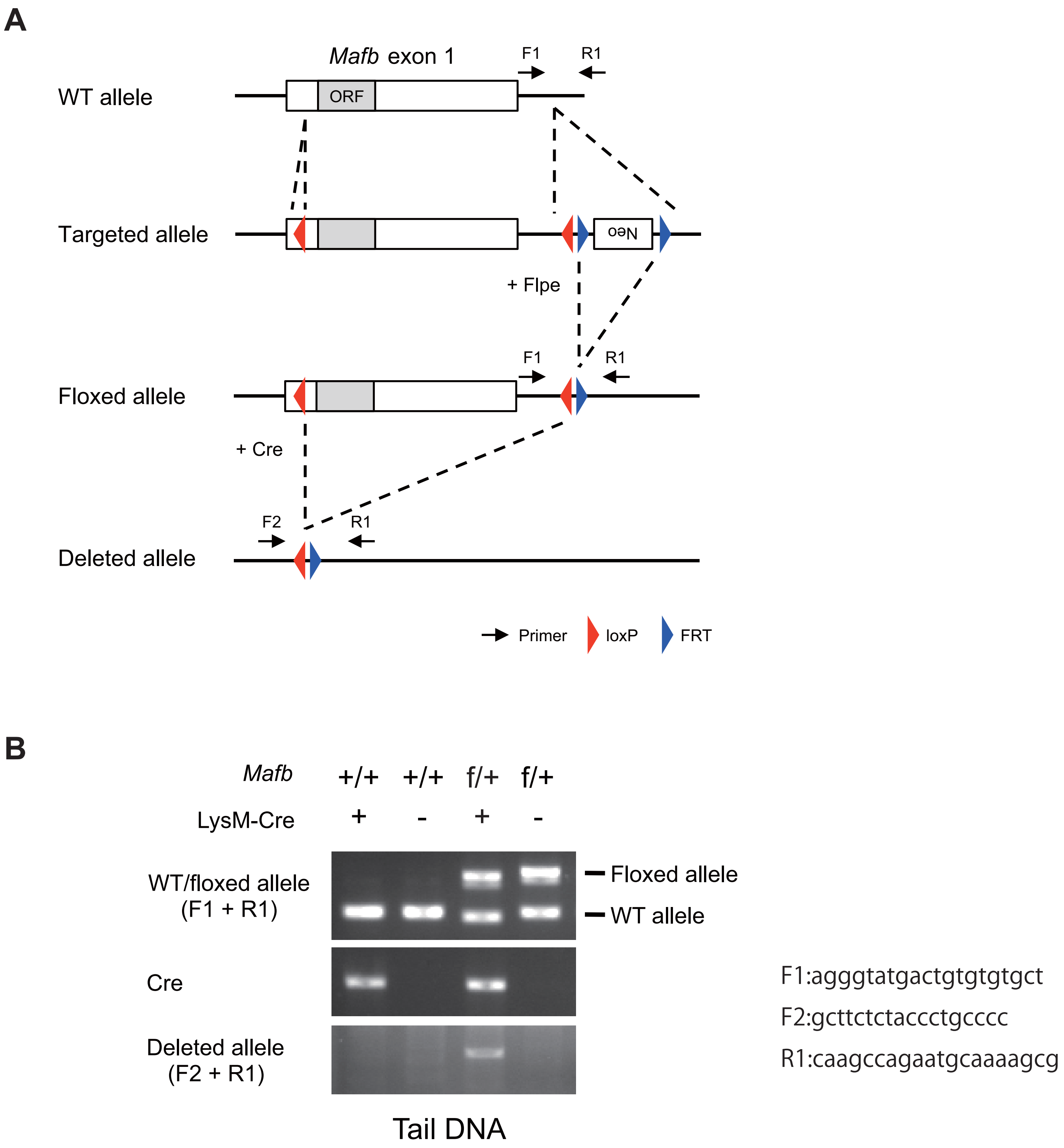

Supplementary Fig. 6. Generation of *Mafb* conditional knock-out mice.
(**A**) Strategy for the generation of *Mafb* conditional knock-out mice. The *Mafb* gene was flanked by loxP elements, the neomycin-resistance gene (NEO) was flanked by FRT elements, and the NEO cassette was deleted through mating with flippase (FLP)-expressing mice. F1, F2, and R1 indicate the genotyping PCR primers. (**B**)Genotyping PCR results. The upper panel shows the PCR results using F1 and R1. The Cre recombinase gene was detected and is shown in the middle panel. PCR using F2 and F1 detected a deleted allele (lower panel). The genotyping primers were F1:5'-agggtatgactgtgtgtgct-3', F2: 5'-gcttctctaccctgcccc-3', and R1:5'-caagccagaatgcaaaagcg-3'.

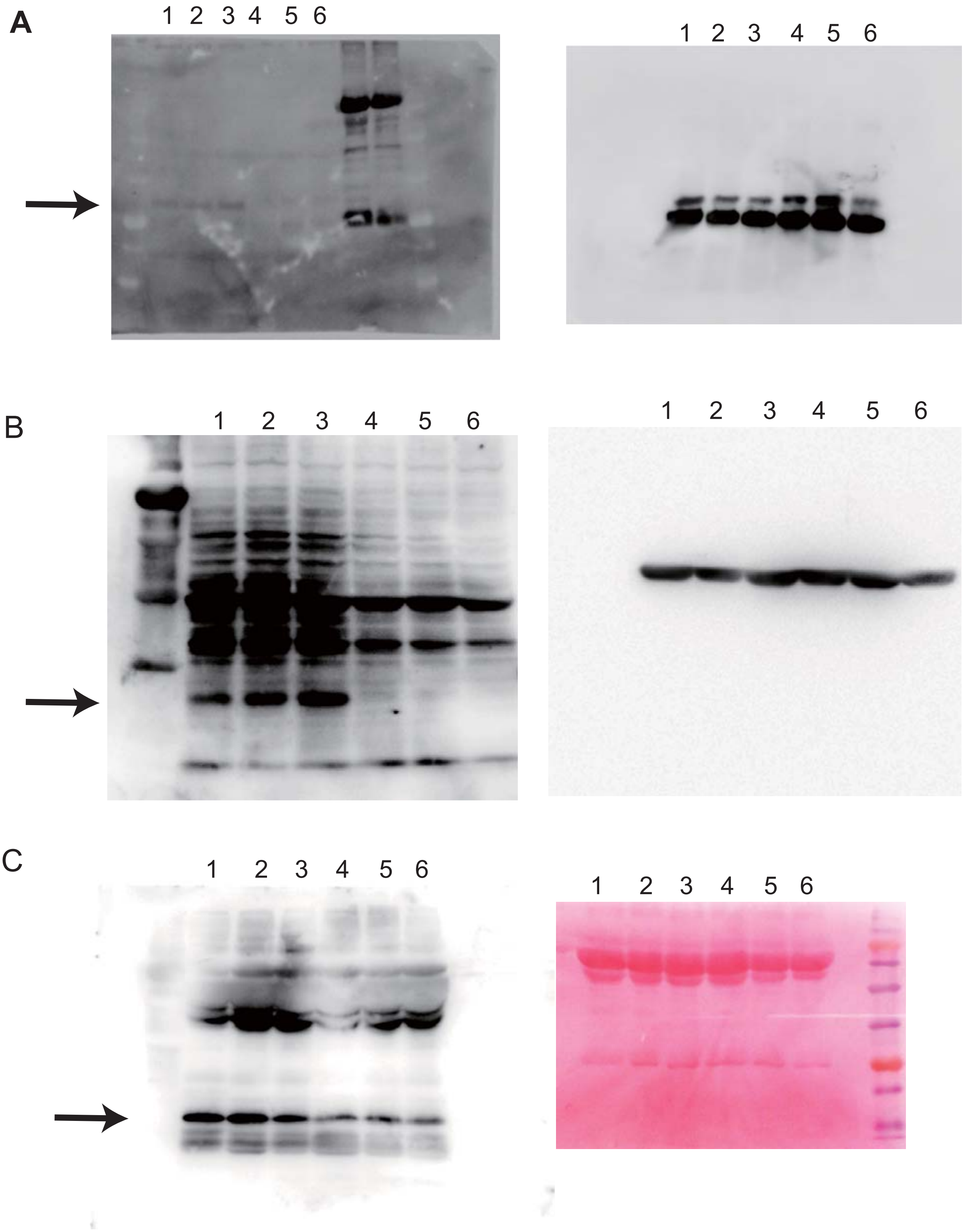

Supplementary Fig. 7 Image of the full western blots in Figure 2.
(A) Full blot image of Fig. 2C, upper panel. An anti-C1q antibody was used in the right panel, and an anti-beta-actin antibody was used in the left panel. Lanes 1,2 and 3 are WT , 3,4, and 6 are *Mafb*$^{-/-}$ macrophages.(B) Full blot image of Fig. 2C, lower panel. Lanes 1, 2 and 3 are *Mafb*$^{f/f}$ macrophages, and lanes, 4, 5 and 6 are *Mafb*$^{f/f}$*::LysM-Cre* macrophages. An anti-C1q antibody was used in the right panel and an anti-beta-actin antibody was used in the left panel. (C) Full blot image of Fig. 2G. An anti-C1q antibody was used in the right panel. The left panel shows the full image of ponceau S staining. Arrows indicates the C1q band (approximately 30 kDa).

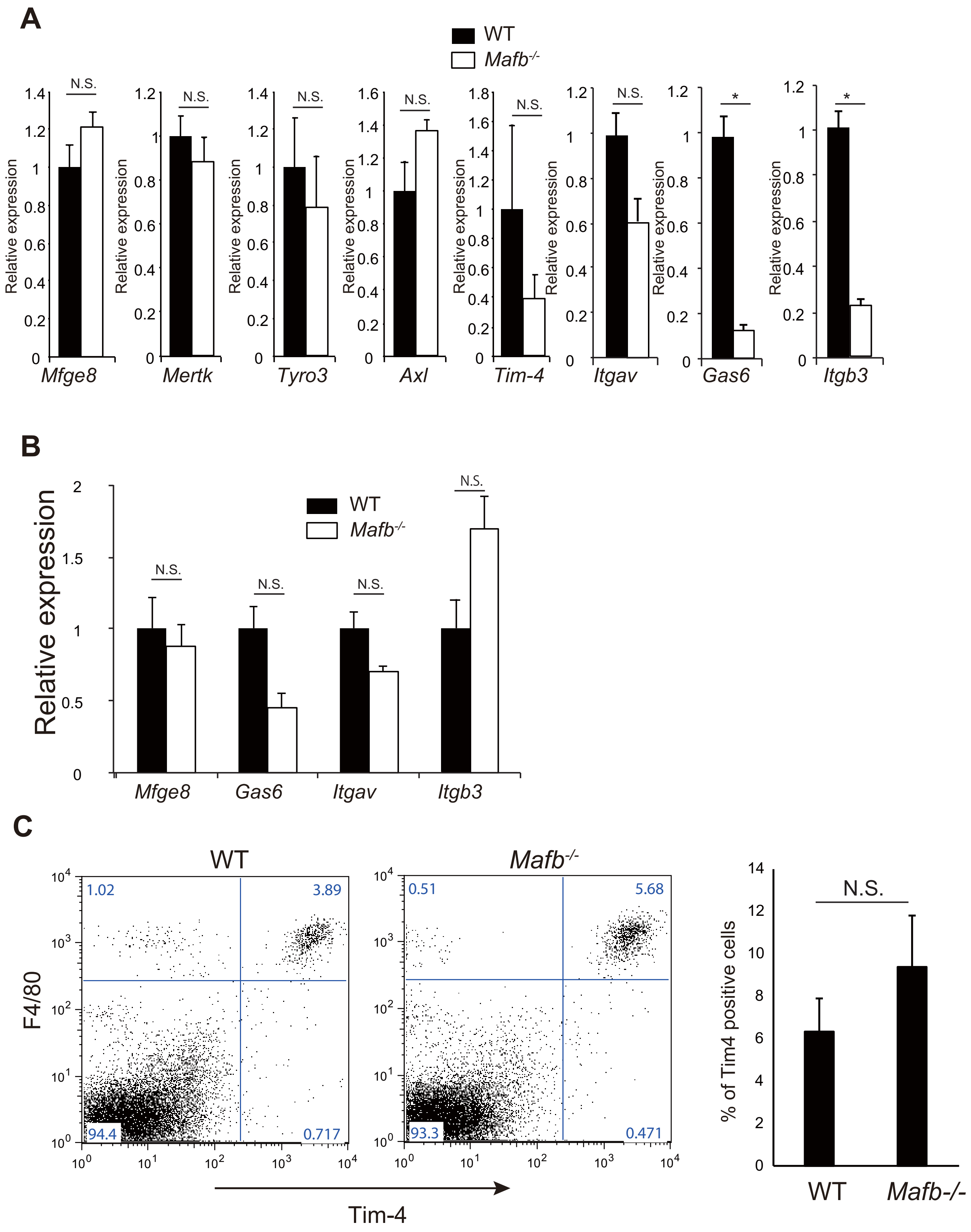

Supplementary Fig. 8. Expression of apoptotic recognition factors did not differ between WT and *Mafb*$^{-/-}$ in macrophages.

(**A**) mRNA levels of opsonins and receptors in WT and *Mafb*$^{-/-}$ macrophages from E17.5 fetal livers were measured by qRT-PCR (WT, n = 3; *Mafb*$^{-/-}$, n = 3). (**B**) mRNA levels of opsonins and receptors in peritoneal macrophages were examined by qRT-PCR (WT, n = 3; *Mafb*$^{-/-}$, n = 3). (**C**) FACS analysis showed that Tim4 expression was detected in F4/80+ resident peritoneal macrophages from both WT and *Mafb*$^{-/-}$ mice (n = 3 for each group). The right panel showes the statistical analysis which revealed no difference between WT and *Mafb*$^{-/-}$. The data are from one experiment that is representative of two independent experiments. (**A**, **B**, **C**) Data are presented as the mean ± s.e.m.; *, $p < 0.05$; N.S., not significant (Welch's t-test).

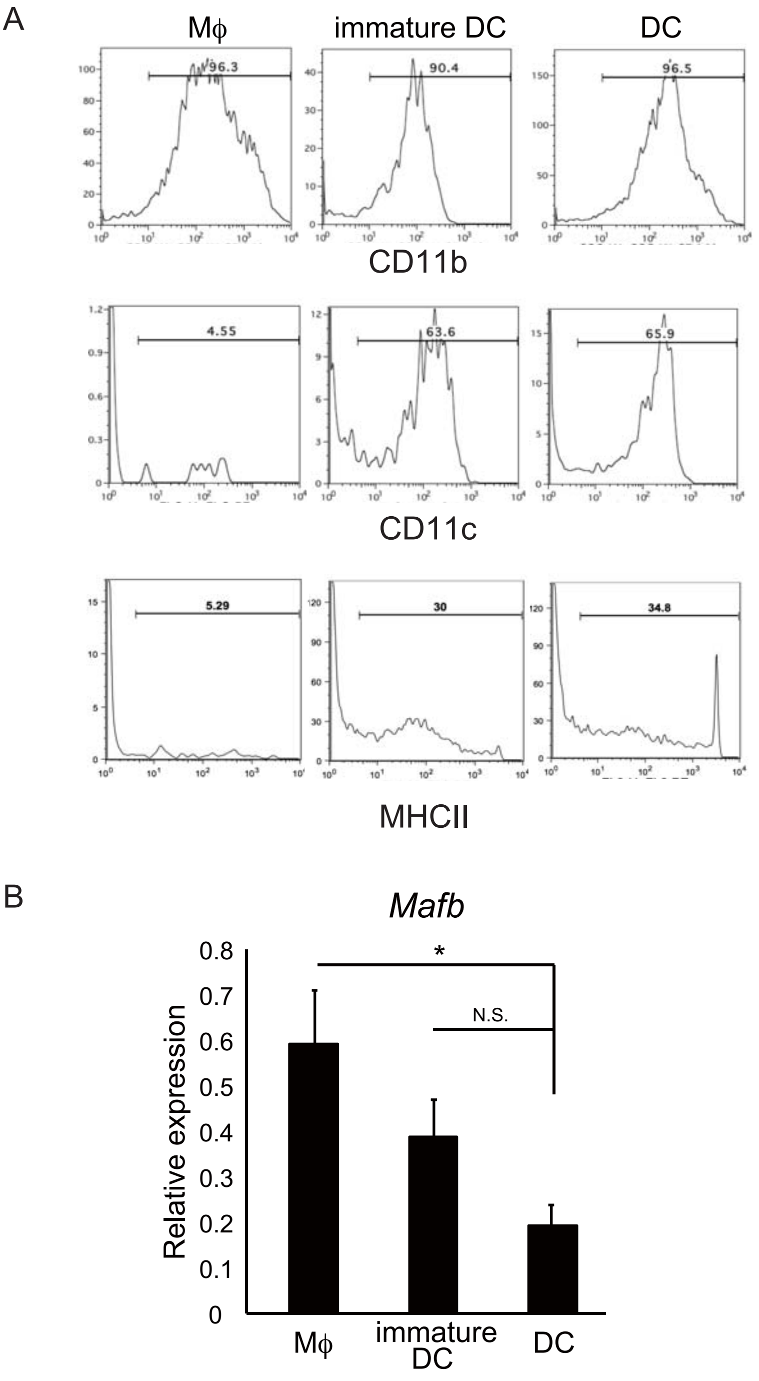

Supplementary Fig. 9. Induction of DCs and the examination of *Mafb* expression in DCs.

(**A**) The FACS analysis showed the expression of CD11b, CD11c, and MHCII in M-CSF induced macrophages; IL4- and GM-CSF-induced immature DCs, and IL4-, GM-CSF- and LPS-induced DCs from WT mice. (**B**) *Mafb* expression was examined by qRT-PCR analysis of macrophages, immature DCs and DCs. The data were normalized to *Hprt* and the results are presented as the mean +s.e.m.; *, $p < 0.05$; N.S., not significant (Student's t-test). (**B**) n = 4 for each group. The data were normalized to *Hprt*, and the results are presented as the mean +s.e.m.; *, $p < 0.05$; N.S., not significant (Student's t-test).The data are from one experiment that is representative of at least two independent experiments.

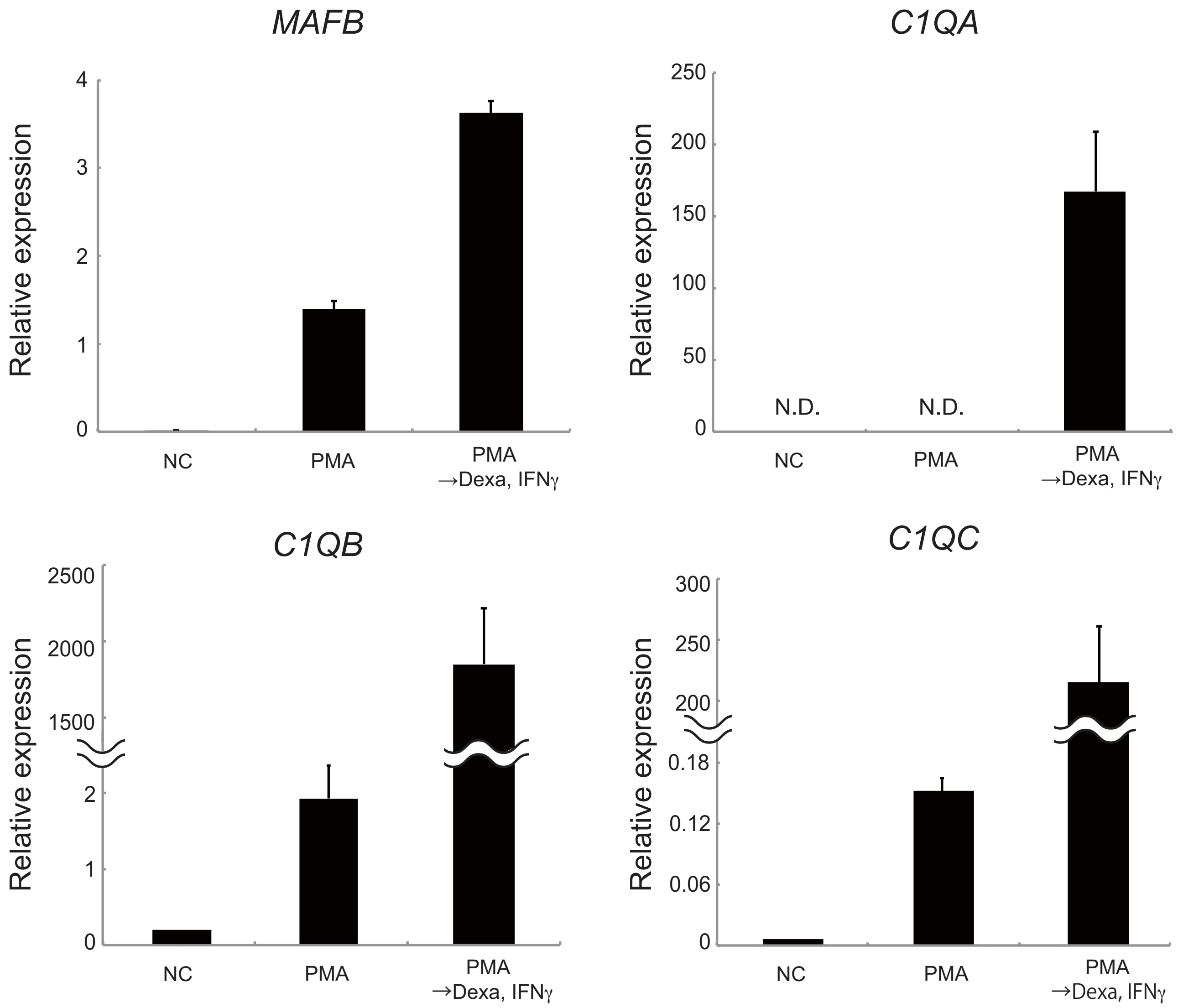

Supplementary Fig. 10. *Mafb* and *C1q* gene expression in THP-1 cells.
qRT-PCR analysis of *MAFB*, *C1QA, C1QB,* and *C1QC* expression in THP-1 cells treated with or without PMA or with PMA, dexamethasone and IFNγ. *MAFB, C1QA, C1QB,* and *C1QC* were strongly induced by dexamethasone and IFNγ. The data are from one experiment that is representative of at least three experiments. The data were normalized to *HPRT,* and the results are presented as the mean ± s.e.m. of duplicate samples. The data are from one experiment that is representative of two independent experiments performed in duplicate.

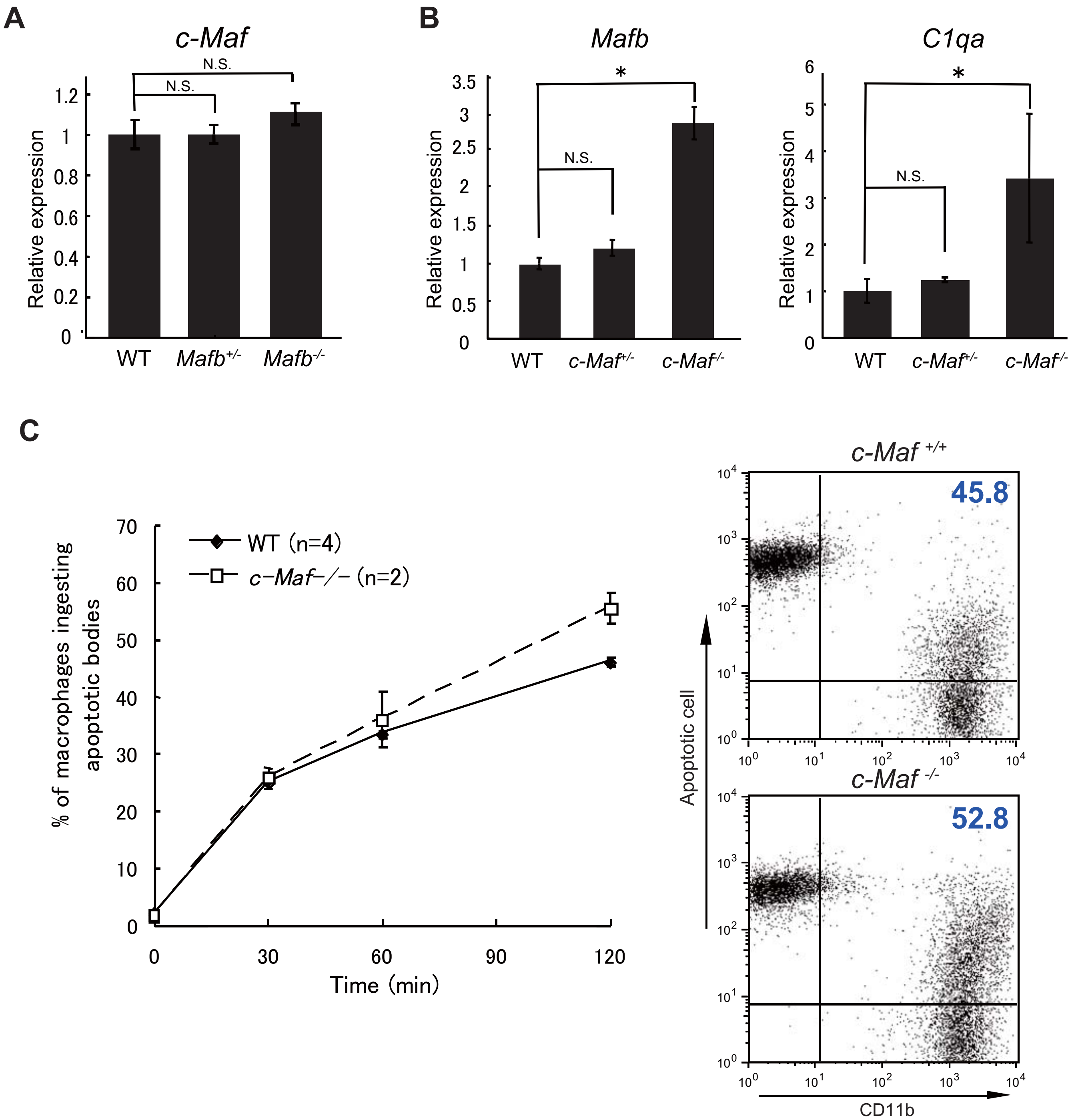

Supplementary Fig. 11. c-Maf is not related to *C1qa* gene expression.
(**A**) qRT-PCR analysis of *c-Maf* expression in WT, *Mafb*$^{+/-}$, and *Mafb*$^{-/-}$ of fetal liver derived macrophages (n = 4 for each group). (**B**) qRT-PCR analysis of *Mafb* and *C1qa* expression in WT, *c-Maf* $^{+/-}$, and *c-Maf* $^{-/-}$ of fetal liver-derived macrophages (n = 4 for each group). (**C**) Left panel: Apoptotic Jurkat cells were incubated with WT and *c-Maf* $^{-/-}$ macrophages. The percentage of binding or uptake of apoptotic cells was increased in a time-dependent manner. Right panel: FACS plot at 120 min after incubation. (**A, B**) Data were normalized by *Hprt,* and the results are presented as the means ± s.e.m.; *, $p < 0.05$; N.S., not significant (Student's t-test). The data are from one experiment that is representative of at least two independent experiments.

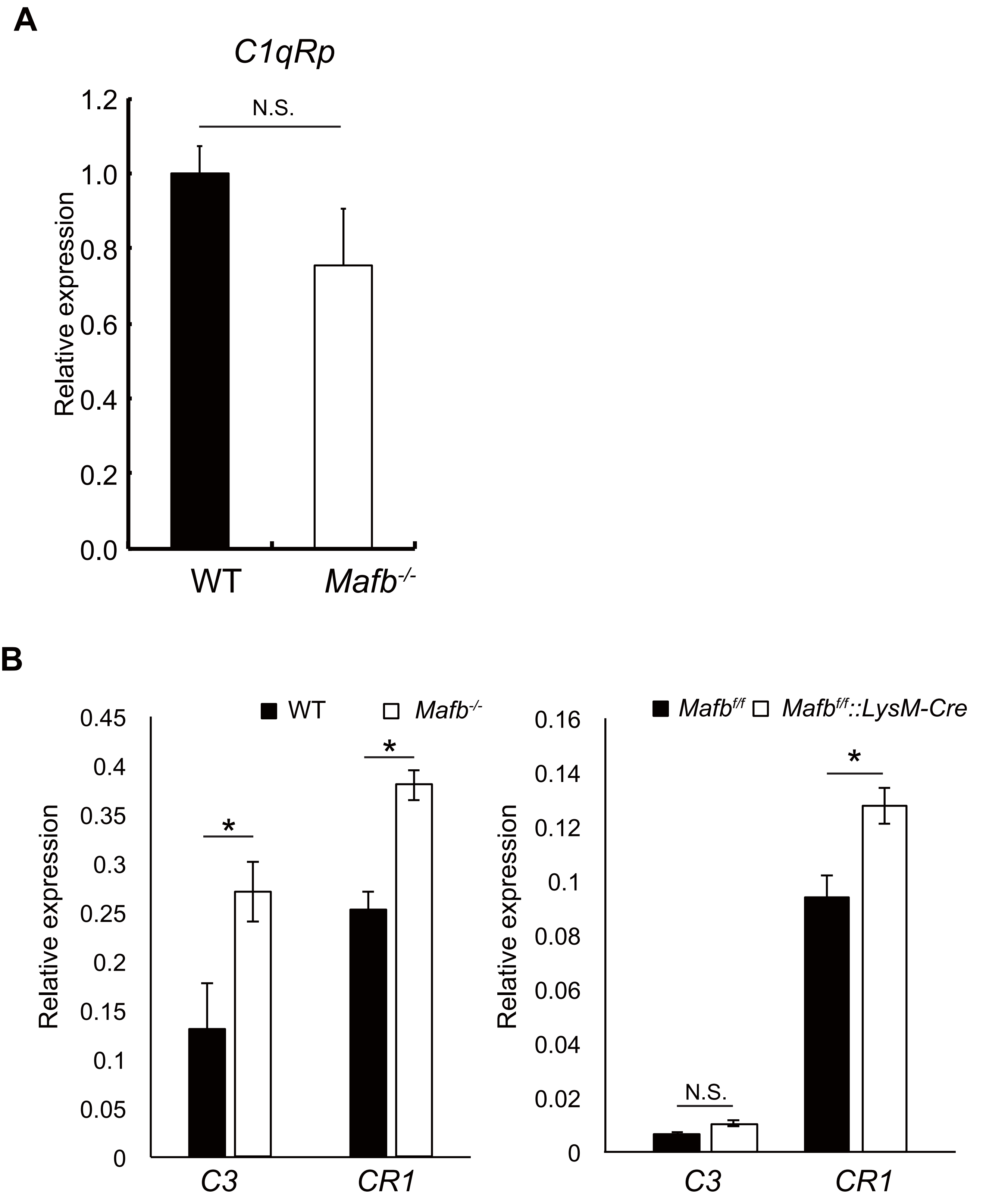

Supplementary Fig. 12. *C3, CR1*, and *C1qRp* expression in *Mafb* deficient and control macrophages.

(**A**) *C1qRp* expression was analyzed in WT and *Mafb*$^{-/-}$ fetal liver derived macrophages (n=5 for each group). (**B**) qRT-PCR analysis of *C3* and *CR1* expression in WT, *Mafb*$^{-/-}$, *Mafb*$^{f/f}$ and *Maf*$^{f/f}$::*LysM-Cre* bone marrow-derived macrophages (n=4 for each group). (A, B) Data were normalized to *Hprt* expression, and the results are presented as the mean ± s.e.m.; *, $p < 0.05$; N.S., not significant (Student's t-test). The data are from one experiment that is representative of at least two independent experiments

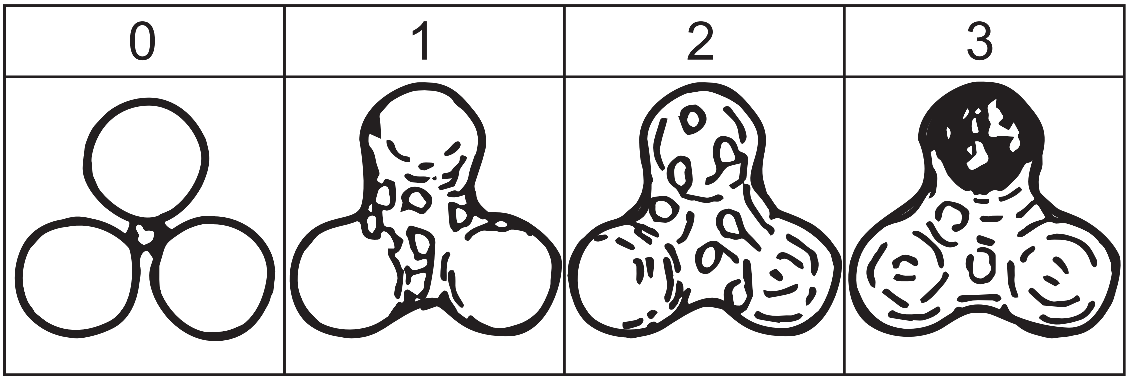

Supplementary Fig. 13. Semiquantitative analysis of glomerular lesions in WT and *Mafb*$^{-/-}$ mice

The images show capillary vessels in glomeruli. The degree of severity was estimated on a scale of 0 to 3: 0, normal architecture; 1, increased mesangial substrate and numerous infiltrating cells between capillaries; 2, increased cell infiltration in capillary vessels; and 3, disrupted glomeruli structure.

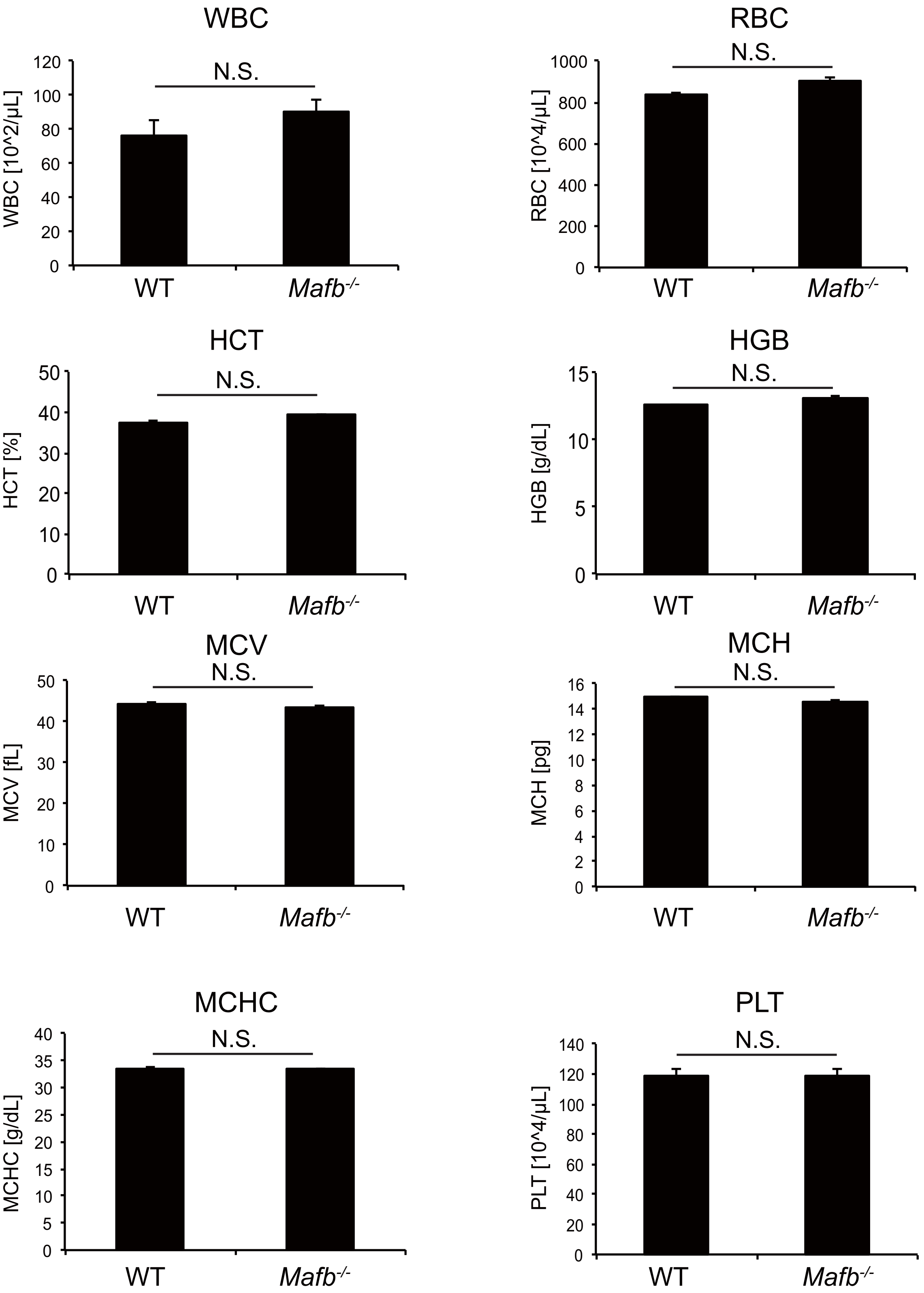

Supplementary Fig. 14. Hematological analysis of peripheral blood, spleen, and bone marrow cells from recipient mice transplanted with fetal liver cells from WT and *Mafb*$^{-/-}$ mice.
Peripheral blood samples from WT and *Mafb*$^{-/-}$ mice (n = 6 for each genotype) were analyzed with the automatic hematology analyzer Celltac-α. WBC, white blood cell; RBC, red blood cell; MCV, mean corpuscular volume; MCH, mean corpuscular hemoglobin; HGB, hemoglobin; HCT, hematocrit; MCHC, mean corpuscular hemoglobin concentration; and PLT, platelet. Data are presented as the mean + s.e.m.; N.S., not significant (Student's t-test).

Supplementary Table 1. Primer sequences used for all experiment

qRT-PCR	Sense	Anti-sense
**Mouse**		
Mafb	TGAATTTGCTGGCACTGCTG	AAGCACCATGCGGTTCATACA
C1qa	GGATGGGGCTCCAGGAAATC	CTGATATTGCCTGGATTGCC
C1qb	TGGCTCTGATGGCCAACCAG	GACTTTCTGTGTAGCCCCGT
C1qc	AGGACGGGCATGATGGACTC	TGAATACCGACTGGTGCTTC
PPARδ	GAGGGGTGCAAGGGCTTCTT	CGCCACCAGCTTCCTCTTCT
Mac-1	ATGGACGCTGATGGCAATACC	TCCCCATTCACGTCTCCCA
Tim4	CACAAAAGGGTCCGCCTTCA	CCTGAGTACGGCTATGTCTG
Mfge8	GATCTTTCCAACAACCTAGCCTCC	ACCGCTTTCATCCTGGATGAACTC
Gas6	GCCCCTCTCCAGCTGAAACA	GTGGCAAACTATCTCCGTGG
ItgαV	CGCCTATCTTCGGGATGAATC	CCAACCGATACTCCATGAAAAATG
Itgβ3	ATGTGTTCCGGCCATGGGCA	AGCCCATTGGTGGACATGCA
Mertk	CCTGAACACAGTAAGGTAGA	ATGAATCCACAGAAGCAGCC
Tyro3	GTACTGTACAGGTGGCACAC	ATTGGCACAGCGCACCCTAA
Axl	TACCGGCTGGCATATCGAGG	AGACACAGTCAGGTTAGCCA
Hprt	CAAACTTTGCTTTCCCTGGT	CAAGGGCATATCCAACAACA
Beta-actin	TGTATGAAGGCTTTGGTCTCCCT	AGGTGTGCACTTTTATTGGTCTCAA
**Human**		
hMAFB	TGAAATTGCTGGCGCTGCTG	AAGCACCATGCGGTTCATACA
hC1QA	ATGGTGACCGAGGACTTGTG	GTCCTTGATGTTTCCTGGGC
hC1QB	GGCTTCCAGGGCTGGCTGGAG	TCCCGATTCACCTTTGGGGCC
hC1QC	GAATCCCAGCCATTCCCGGGA	GCCCTCCTCACCTGGCTCTCC
hHPRT	TCATGGACTAATTATGGACAGGACT	CAGCAGGTCAGCAAAGAATTTATAG

ChIP assay	Sense	Anti-sense
**Mouse**		
C1qa MARE	GCTTTCTGGATCCTGAAGAACCC	GACTTCCCCTTCTTGGAATCTACC
C1qb MARE	GGCAAGCAGCCATCTGGGGATAGT	TCTCTATCACCCCTCTGTTCCTCCC
C1qc MARE	GCACACATGACCTAGGGCCAGGGAA	GAGACGTCCTGTTCTCCCAGACAGG
**Human**		
hGAPDH promoter	TACTAGCGGTTTTACGGGCG	TCGAACAGGAGGAGCAGAGAGCGA
hC1qa MARE1	ATCCTCACAGGCACTGGCCT	TCAGACCTGCCCCCTGAACT
hC1qa MARE2	GAGCTGCCCTCAGTTTCCCC	CCCTCCTCTTGGCGCCCACT
hC1qb MARE	CCCCTGCCCAGTGTAGAAGA	ATGAGACGGGTGGGTGGCAA
hC1qc MARE	TCCACCTCCAAGTGTGGCCA	GGAGGATGTACACCGTTGCC

Mutation construct	Sense	Anti-sense
**Mouse**		
C1qa mut-MARE	CCAAACGTCGCGAGGTCCGCTTAA	TTAAGCGGACCTCGCGACGTTTGG
C1qb mut-MARE	CACAGGTCGCGAGTTGGCGAAACCAGGC	GATGGGGTTGGTGGTGGGAGGTCGGG
C1qc mut1-MARE	GTGGTCGCGCTGGATCAGAACCTAAAA	AGTGATATCTCTACCCACCCCTTCCTT
C1qc mut2-MARE	ATGTAGGGTTAGGGTCGCGTGGGG	CCCCTTTCCAGAAATTTTCCCTGACTT
